# Seasonal Changes in the Biochemical Constituents of Green Seaweed *Chaetomorpha antennina* from Covelong, India

**DOI:** 10.3390/biom12101475

**Published:** 2022-10-13

**Authors:** A. Vinuganesh, Amit Kumar, Shereen Magdy Korany, Emad A. Alsherif, Samy Selim, Sanjeevi Prakash, Gerrit T. S. Beemster, Hamada AbdElgawad

**Affiliations:** 1 Centre for Climate Change Studies, Sathyabama Institute of Science and Technology, Chennai 600119, India; 2 Department of Biology, College of Science, Princess Nourah bint Abdulrahman University, Riyadh 11671, Saudi Arabia; 3 Biology Department, College of Science and Arts at Khulis, University of Jeddah, Jeddah 21959, Saudi Arabia; 4 Botany and Microbiology Department, Faculty of Science, Beni-Suef University, Beni-Suef 62521, Egypt; 5 Department of Clinical Laboratory Sciences, College of Applied Medical Sciences, Jouf University, Sakaka 72388, Saudi Arabia; 6 Laboratory for Molecular Plant Physiology and Biotechnology, Department of Biology, University of Antwerp, Groenenborgerlaan 171, B-2020 Antwerp, Belgium

**Keywords:** seasonality, tropical seaweed, oxidative stress, nutritional quality, minerals

## Abstract

Seaweeds are well known for having a wealth of nutritional benefits and providing ecological support to associated fauna. Seasonality influences the biochemical characteristics, affecting their ecological and economic values. In the present study, we evaluated pigments, primary and secondary metabolites, minerals, and antioxidant properties of green seaweed *Chaetomorpha antennina* growing on the intertidal rocks along the Covelong coast, India, in different seasons (from June 2019 to March 2020). Significant variations were found in the levels of antioxidants, minerals, and metabolites in different seasons, e.g., amino acid levels were the highest in post-monsoon and the lowest in summer. In monsoon, we found the highest concentration of fatty acids in the thalli. Lipid peroxidation and total antioxidant activity were at their maximum levels during post-monsoon, which indicated oxidative damage responses. No significant variations were found in the levels of photosynthetic pigments. The outcomes indeed suggested seasonal variations in the biochemical and nutrient profile of *C. antennina*. We suggest that the harvesting/collection of *C. antennina* for different nutrients and metabolites should be performed in the respective seasons.

## 1. Introduction

Seaweeds play a vital role in the marine ecosystem, as they form the base of the coastal and marine food web [1]. Seaweeds perform several ecological services, such as providing habitat, feeding, and breeding grounds to numerous marine flora and fauna [2,3]. They are involved in nutrient recycling [4], the bioremediation/phytoremediation of harmful pollutants, etc. [5,6]. Seaweeds are an economically important group of organisms; they are used as a source in many industrial applications, such as for food, fodders, fertilizers, biofuel, nutraceuticals, etc. [7]. Many types of seaweed pose nutraceutical properties and protect against various neurological disorders in humans [2,8,9].

Benthic seaweeds growing in the intertidal zone experience a wide range of abiotic and biotic stresses, including strong fluctuations in tides, photoperiod, temperature, salinity, and nutrients; strong wave action; desiccation; ultraviolet radiations; and pollutants [1,10,11,12]. Seaweeds have adapted to survive these harsh conditions by regulating their cellular mechanisms, such as undergoing osmotic adjustments and metabolic processes [10]. The vertical zonation on the intertidal rocks is also determined by the tolerance of the species to abiotic factors [13]. The environmental parameters determine the photo-physiological performance and biochemical composition of seaweeds [14]. The biotic and abiotic interactions vary according to the seasons, leading to the changes in ecological conditions that can stimulate or inhibit the uptake of minerals, and in the biosynthesis of primary and secondary metabolites [15]. The changes in these metabolites may affect the nutritional quality of seaweeds, thereby impacting their ecological services to the associated faunal communities [16]. Additionally, changes in these metabolites and mineral content can affect their commercial applications [17]. Due to seasonal fluctuations, seaweeds can change their biochemical constituents, including the levels of sugars, amino acids, fatty acids, minerals, etc. These changes could be a possible indicator of the physiological state of seaweeds. Seasonal changes in biochemical compositions are also influenced by physiological changes within vegetative thalli [18]. These stresses commonly induce oxidative stress (production of reactive oxygen species (ROS)), which affects the integrity of biomolecules through lipid peroxidation, the oxidation of proteins, and the damaging of nucleic acids [19]. To counter ROS, seaweeds have diverse antioxidant enzymes, such as superoxide dismutase (SOD) and catalase (CAT), the ascorbate-glutathione pathway, and non-enzymatic molecules such as polyphenol, carotenoids, phenols, ascorbate, glutathione (GSH), tocopherols, and mycosporine-like amino acids [20,21,22,23,24]. Previous studies on seasonality indicate the fluctuations in the biochemical composition and oxidative stress of green algae [8,25,26,27,28], red algae [25,26,27], and brown algae [28,29,30,31].

Light is the strongest factor that affects the photosynthetic apparatus in algae, which alters the concentration and compositions of pigments [12]. Photosynthetic pigments (chlorophyll and carotenoids) are involved in the growth and development of seaweeds, as they utilize solar energy and convert it into carbohydrates through photosynthesis. The produced energy is utilized or transferred to the next trophic level in the food web/ food chain [8]. The variations in temperature during different seasons may alter the levels of metabolites in seaweeds. Temperature affects the membrane fluidity in seaweeds; as a consequence, it may alter their fatty acid composition. At increased temperatures, seaweeds maintain their fluidity by minimizing the concentration of polyunsaturated fatty acids (PUFAs) and saturated fatty acids (SFAs) [2]. These influences of various abiotic factors may affect the nutritional quality of seaweed and thus the ecological and commercial aspects of seaweeds [2]. Therefore, it is necessary to understand seaweed physiology in various seasons.

The *Chaetomorpha* genus is represented by more than hundreds of species, widely distributed in the intertidal and sub-tidal zones across the global ocean [11,32,33]. Some *Chaetomorpha* species are proven to have commercial potential as a feedstock for bioethanol production [34,35]. *C. antennina* is widely available along the coastlines of India [36,37,38,39]. Though the species is not listed as Generally Regarded to be Safe (GRAS) for consumption, the recent literature reports its potential in various applications. Metabolites obtained from them have been evaluated in light of their commercial applications, including antioxidant and antidiabetic activities, bioaccumulation of minerals [40,41,42,43]. Since the biochemical characteristics and metabolites may vary seasonally and no information exists in this regard, we attempted to understand these aspects in the present study. The objective of the present study was to establish the nutraceutical importance of green alga *C. antennina* in terms of its nutritional and antioxidant properties and also to determine the apt period for harvesting based on the nutritional requirements for commercial utilizations. In the current study, we looked into temporal variations in the biochemical constituents, such as pigments, metabolites (primary and secondary), oxidative status, minerals, etc., of rocky intertidal green seaweed *Chaetomorpha antennina*, from Covelong, Chennai, India.

## 2. Materials and Methods

### 2.1. Sampling Site and Collection

*Chaetomorpha antennina* were collected on the rocky intertidal coast of Covelong (12°47′31″ N; 80°15′04″ E–12°46′42″ N; 80°15′15″ E), located 40 km south of Chennai, India, every month between June 2019 and March 2020. The samples were not collected during April and May 2020 because of the unfavorable situation due to the COVID-19 lockdown. Nevertheless, our samples represented the overall seasonal variation. The algal thalli were taken in triplicates from three intertidal rocks situated 100 m apart. The algal sample (~5 gm thalli) was collected by scraping the entire thalli from holdfast using a scalpel. The samples were transported to the laboratory within 30 min of collection, were cleaned for epibiota using soft brushes and sterilized seawater, and were stored in a deep freezer. Standard Keys were used to identify taxa at the microscopic level [44,45,46]. One algal thallus from each rock (so, 3 samples every month) was used for further analyses. We considered three seasons: summer (June–September), monsoon (October–December), and post-monsoon (January–March) based on the temperature and rainfall in this area. Water quality parameter at the time of sample collection in the study site is shown in Supplementary Table S1.

### 2.2. Photosynthetic Pigments

Chlorophyll *a* and *b*, and carotenoids were estimated as described by Vinuganesh et al. [24]. Briefly, 50 mg fresh weight of samples was used for the preparation of seaweed extract using 90% acetone. The extract was incubated at 4 °C overnight in the dark. The extract was centrifuged at 5000 rpm for 5 min at 4 °C, and the supernatants were collected. The supernatants were measured at 664, 647, and 470 nm using a spectrophotometer (JASCO V670, Tokyo, Japan). The concentrations of photosynthetic pigments were measured using the equation provided by Jeffrey, Humphrey, and Lichtenthaler [47]. The concentration was expressed as microgram per gram of fresh weight (µg/g FW).

Chlorophyll *a* = 11.93 E_664_ − 1.93 E_647_ (1)

Chlorophyll *b* = 20.36 E_647_ − 5.50 E_664_ (2)

Carotenoids = ((1000 × A470) − (1.82 × Chl*a*) − (85.02 × Chl*b*))/198 (3)

### 2.3. Metabolite Analysis

Carbohydrates were estimated in acetonitrile/water (2 mL, 1:1, *v*/*v*) extract and quantified using high-performance liquid chromatography (HPLC) according to Alasalvar et al. [48]. The prepared extract was incubated at 55–60 °C in a water bath for 15 min and filtered using Whatman No. 541 filter paper; after it was brought to a final volume of 100 mL using extraction solvent, it was analyzed using HPLC in a 5 µm SUPELCOSIL LC-NH2 column (250 × 4.6 mm) at a temperature of 30 °C and eluted with acetonitrile-HPLC-grade water (75:25 *v*/*v*). Individual carbohydrates were quantified by comparing standard curves obtained using known concentrations of standard sugar solutions ranging from 1 to 10 mg/100 mL of acetonitrile/water (1:1, *v*/*v*). QA/QC was ensured with the standard addition of sugars to ensure the best recoveries. The limit of detection (LOD) was 5.2 ng; the limit of quantification (LOQ) was 22 ng; R2 values were 0.993 and 1.00. The correlation coefficient (R2) was in the range of 0.991–0.998.

Seaweed fatty acids (FAs) were estimated using Gas Chromatography/Mass Spectrometer using aqueous methanolic extract (1:1 *w*/*v*) until discoloration happened (see Torras-Claveria et al. [49]). Codeine and non-adecanoic acids were used as standards. Seaweed extract was quantified for available FAs with GC/MS using a Hewlett Packard 6890, MSD 5975 mass spectrometer (Hewlett Packard, Palo Alto, CA, USA), with an HP-5 MS column (30 mm × 0.25 mm × 0.25 mm). Individual FAs were identified with the NIST 05 database and plant-specific databases (e.g., GolmMetabolome Database, http://gmd.mpimp-golm.mpg.de/, accessed on 10 December 2021). The recovery percentages (R%) values for plant samples ranged from 91.6 to 98.2%. The correlation coefficient (R2) was in the range of 0.997–1.0000. The limit of detection (LOD) was 2.7 ng; the limit of quantification (LOQ) was 34 ng; R2 values were 0.995 and 1.00.

Seaweed amino acids (AAs) were estimated using I mL of 80% (*v*/*v*) aqueous ethanolic extract. The extract was centrifuged, and the supernatant was evaporated under vacuum. The pellet was dissolved in 1 mL of chloroform, and the suspension was re-extracted using 1 mL of HPLC-grade water. Then, the aqueous phase was collected after centrifugation and was filtered using 0.2 μM Millipore microfilters. AAs were analyzed using a Waters Acquity UPLC-tqd system (Milford, Worcester County, MA, USA) equipped with BEH amide 2.1 × 50 columns. QA/QC was ensured with the standard addition of amino acids to ensure the best recoveries. The method limits of quantification (MLQs) of most amino acids, determined using the concentration obtained via the method for preconcentration under reduced pressure in ultrapure water, were 0.1–40 μg L^−1^. The average recovery was 94% for most of the amino acids.

### 2.4. Redox State Estimation

Lipid peroxidation was measured by analyzing malondialdehyde (MDA) according to Hodges et al. [50]. A ferric reducing/antioxidant power (FRAP) assay was employed for measuring the total antioxidant capacity according to Benzie and Strain [51]. Ascorbate (ASC) and glutathione (GSH) were estimated using seaweed extract prepared using 6 % ice-cold metaphosphoric acid using a reversed-phase HPLC column (100 × 4.6 mm; Polaris C18-A; 3 µm particle size) at 40 °C with an isocratic flow rate of 1 mL min-1 of elution buffer (2 mMKCl; pH 2.5 adjusted with o-phosphoric acid). Concentrations of total ASC (ASCt) and GSH (GSHt) (reduced + oxidized) were estimated according to Potters et al. [52]. Total polyphenols and flavonoids were measured using 80 % ethanolic extract. The Folin–Ciocalteu method was adopted and gallic acid was used as a standard for measuring the total phenols based on Zhang et al. [53]. The modified aluminum chloride method was adopted, and quercetin was used as a standard for the estimation of flavonoids based on Chang et al. [54]. Tocopherols were measured using hexane extract quantified using HPLC analysis based on Siebert [55]. Data were analyzed with Shimadzu Class VP 6.14 software provided with the HPLC system (Shimadzu, Tokyo, Japan).

Antioxidant enzymatic activities were estimated according to Murshed et al. [56] using seaweed protein extracts. Protein concentration was quantified according to Lowry et al. [57]. Enzyme activities were measured using a microplate reader at 25 °C using 200 µL volume of the reaction mixture. All the enzymatic reactions, such as ascorbate peroxidase (APX), dehydroascorbate reductase (DHAR), monodehydroascorbate reductase (MDHAR), and glutathione reductase (GR), were estimated according to Murshed et al. [56]. Peroxidase (POX) and catalase (CAT) were estimated according to Dhindsa et al. [58] and Aebi [59], respectively. Glutathione peroxidase (GPX) and glutathione S-transferase (GST) activity were estimated according to Drotar et al. [60] and Habig et al. [61], respectively.

### 2.5. Estimation of Minerals

Seaweed samples were digested using HNO_3_/H_2_O (5:1 ratio) in an oven. Macro and trace minerals were determined using mass spectrometry (ICP—MS Finnigan Element XR; Scientific, Bremen, Germany) according to Agusa et al. [62]. Mixtures of standards were prepared in 1% nitric acid.

### 2.6. Statistics

We analyzed the results by combining the months in the season. A one-way ANOVA was performed on the mean values of samples for each season to determine the significant differences using SPSS v21 (SPSS Inc., Chicago, IL, USA). All the statistical analyses considered to have significant positive variations were defined at a significance level of 5% (*p*-value < 0.05). If the F-value showed significance, a comparison was made using post hoc Tukey’s HSD tests. In case the assumptions of the ANOVA were not met, equivalent non-parametric tests were applied. PAST was used for performing a principal component analysis (PCA) of the full dataset. The data were normalized before the PCA by taking the average variable for all months and then taking the difference between each month and the average divided by the standard deviation.

## 3. Results

### 3.1. Photosynthetic Pigments

**Figure 1:**
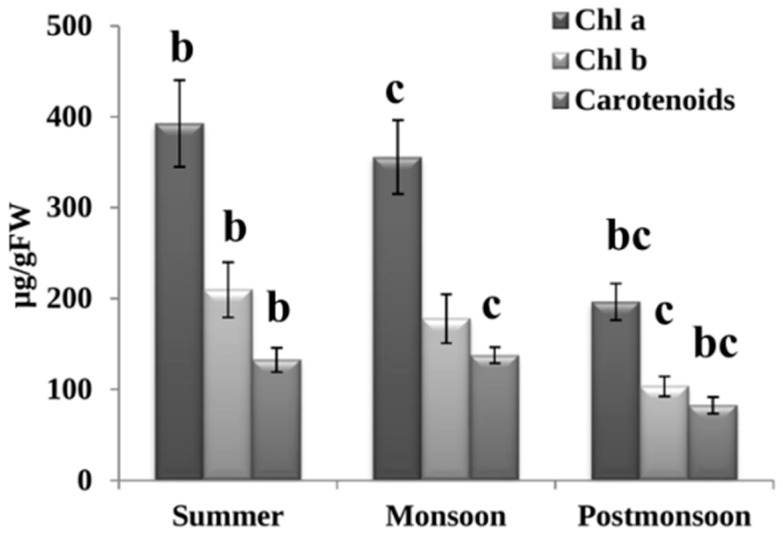
Seasonal variations in the photosynthetic pigments in the tissues of *C. antennina*. Values are shown as means ± S.E. (*n* = 3). Different letters show significance, *p* = 0.05 (b = summer–post-monsoon; c = monsoon–post-monsoon).

We found significant variations in the concentrations of photosynthetic pigments (Figure 1). The highest concentrations of chl *a* and chl *b* (392.5 ± 47.8 µg/g FW and 209.6 ± 30.4 µg/g FW, respectively) were found during summer when compared with post-monsoon (196.4 ± 20.2 µg/g FW and 103.4 ± 10.9 µg/g FW, respectively). While carotenoid levels were higher during monsoon (137.7 ± 8.9 µg/g FW) than during post-monsoon (82.4 ± 9.2 µg/g FW).

### 3.2. Seasonal Variability Affected Primary Metabolites

**Figure 2:**
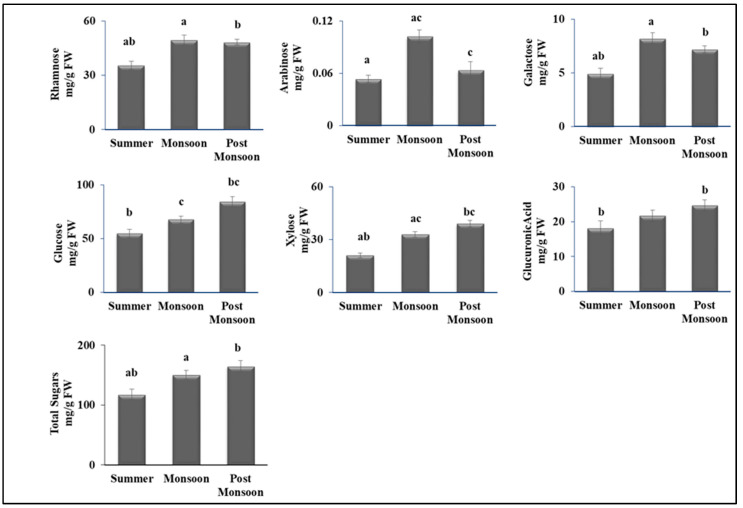
Seasonal variations in various individual monosaccharides and total sugars in *C. antennina*. Values are shown as means ± S.E. (*n* = 3). Different letters show significance, *p* < 0.05 (a = summer–monsoon; b = summer–post-monsoon; c = monsoon–post-monsoon).

Subsequently, we set out to investigate the effects of seasonality on the level of the primary metabolites. A significant increase in total sugars in *C. antennina* was observed during post-monsoon compared with summer. However, the levels of individual monosaccharides such as rhamnose, arabinose, and galactose were high during monsoon compared with summer; glucose and xylose levels were the highest during post-monsoon compared with the other two seasons (Figure 2 & Supplementary Table S2).

**Figure 3:**
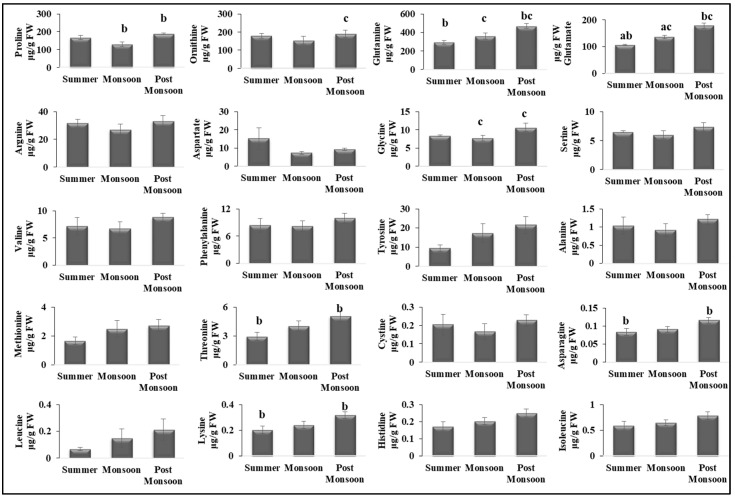
Seasonal variations in the amino acid levels in *C. antennina*. Values are shown as means ± S.E. (*n* = 3). Different letters show significance, *p* < 0.05 (a = summer–monsoon; b = summer–post-monsoon; c = monsoon–post-monsoon).

Then, we investigated the effect on the amino acid levels during different seasons. A total of 20 AAs were identified in *C. antennina*. Proline, glutamine, and glutamate were found in abundance throughout the year (Figure 3 & Supplementary Table S3). These amino acid levels were significantly higher during the post-monsoon season than in summer and monsoon. The levels of other essential amino acids, such as lysine and threonine, and non-essential amino acids, such as glycine and asparagine, were also the highest during the post-monsoon season.

Next, we set out to investigate the fatty acid profile of *C. antennina* during different seasons. A total of 22 fatty acids were identified in *C. antennina*, including 12 saturated fatty acids (SFAs), 4 monounsaturated fatty acids (MUFAs), and 6 polyunsaturated fatty acids (PUFAs) (Table 1). The most abundant fatty acid was hexadecanoic acid (C16:0) throughout the sampling period (Supplementary Table S4). Among the SFAs, the levels of dodecanoic (C12:0), tetradecanoic (C14:0), heptadecanoic (C17:0), octadecanoic (C18:0), docosanoic (C22:0), and pentacosanoic (C25:0) were high during monsoon, and tricosanoic (C23:0), tetracosanoic (C24:0), and hexacosanoic (C26:0) levels were the highest during the post-monsoon season. The concentrations of MUFAs such as hexadecanoic (C16:1) and octadecenoic (18:1) were the highest during monsoon, and that of tetracosenoic (C24:1) was the highest during post-monsoon; PUFAs including hexadecadienoic (C16:2), octadecadienoic (C18:2), octadecatrienoic (C18:3), eicosadienoic (C20:2), and docosenoic (22:2) were at their highest levels during the monsoon period.

### 3.3. Elemental Composition

We also determined the effect of seasonality on the mineral composition of intertidal seaweed *C. antennina*. We found a total of 24 macro and trace minerals in *C. antennina* samples, which are presented in Table 2 & Supplementary Table S5. The post-monsoon period enhanced the concentration of various minerals, such as Na, Rb, Sr, Cd, Cs, Mg, Al, Ca, Mn, Fe, Ga, K, As, and Se, in *C. antennina*.

### 3.4. Redox Status

**Figure 4:**
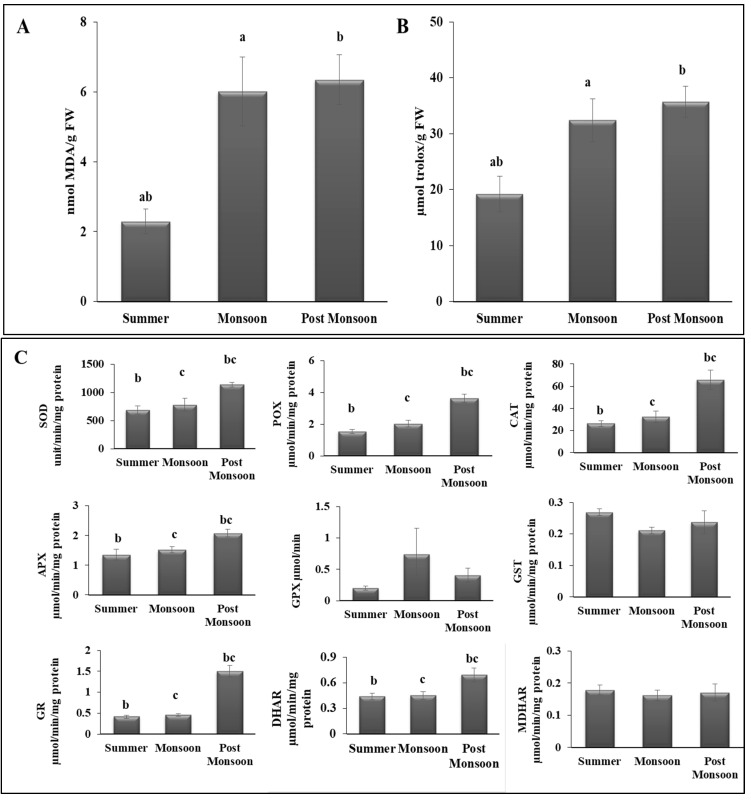

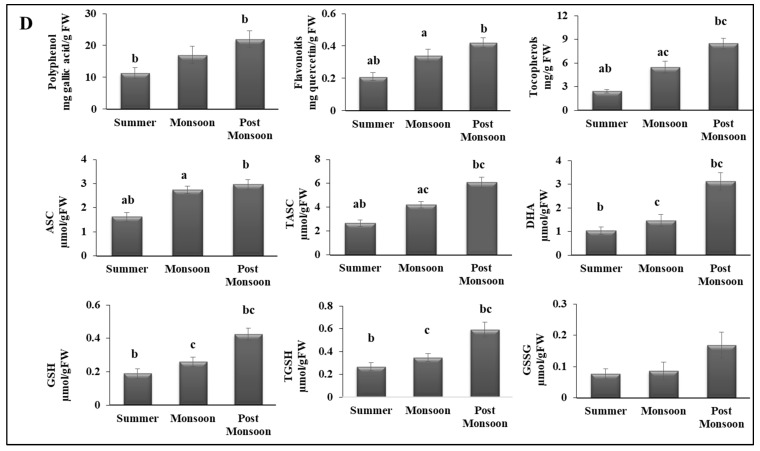
Seasonal variation in lipid peroxidation (**A**) and total antioxidants (**B**), enzymatic (**C**) and non-enzymatic (**D**) antioxidants in *C. antennina*. Different letters show significance, *p* < 0.05 (a = summer–monsoon; b = summer–post-monsoon; c = monsoon–post-monsoon).

Finally, to investigate the protection mechanisms against oxidative stress, we measured lipid peroxidation and total antioxidant capacity. We noticed higher lipid peroxidation during the post-monsoon season (6.35 ± 0.71) than in summer (2.29 ± 0.35) (Figure 4). This indicated oxidative damage during post-monsoon. However, we also found a significant increase in total antioxidants during post-monsoon (35.7 ± 2.76) compared with summer (19.2 ± 3.17) (Figure 4), indicating active oxidative damage response. We also quantified the level of enzymatic and non-enzymatic antioxidant molecules (Supplementary Table S6). We observed that the levels of most of the antioxidant molecules, such as flavonoids, tocopherols, ASC, TASC, DHA, and GSH, and enzymatic antioxidants including GR, DHAR, APX, CAT, POX, and SOD were significantly higher during post-monsoon than in summer and monsoon (Figure 4).

### 3.5. Global Change in the Metabolites and Mineral Composition of C. antennina in Different Seasons

**Figure 5:**
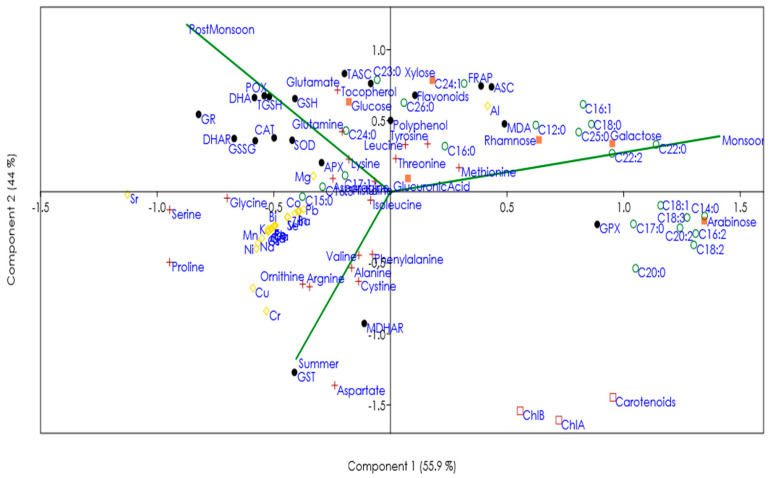
Principal component analysis of seasonal comparison of the levels of antioxidants, metabolites, and minerals in C. antennina. Variances explained by the first two components (PC1 and PC2) appear in parentheses. (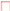 represents pigments, 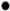 antioxidants, 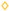 minerals, 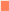 sugars, 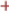 amino acids, and 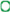 fatty acids).

To obtain a global metabolic view of similarities and differences among the *C. antennina* samples during different seasons, the full dataset was subjected to a principal component analysis (PCA). The first two principal components (PC1 and PC2) accounted for >70% variance (Figure 5). The PCA showed a separation among post-monsoon, summer, and monsoon. FRAP, MDA, antioxidant molecules (including ASC, polyphenols, and flavonoids), sugars (such as xylose, glucuronic acid, rhamnose, and galactose), amino acids (such as methionine, threonine, and tyrosine), fatty acids (such as C12:0, C16:0, C18:0, C22:0, C25:0, C26:0, C16:1, C22:1, and C24:1), and aluminum were positively correlated with the monsoon season.

## 4. Discussion

*Chaetomorpha antennina* is abundantly present on the rocky intertidal shores of Covelong throughout the year, supporting numerous associated invertebrate communities [24,63]. The distribution of *C. antennina* along the rocky intertidal area causes them to be exposed during low tide and submerged during high tide. These tidal variations also have severe implications for light and temperature stresses on seaweeds [8]. The change in environmental parameters may affect the physiology, thereby impacting the biochemical and nutritional properties of the species. Hence, in this study, we sampled algae every month and analyzed the biochemical compositions, antioxidant status, and mineral compositions. These data might help us to understand the effect of seasonality on the commercial importance of nutritional properties such as MUFAs, PUFAs, EAAs, and minerals for harvesting green alga *C. antennina* for commercial applications.

### 4.1. Seasonal Changes Affected Photosynthetic Pigments of C. antennina

In our study, we found that *C*. *antennina* exhibited significant seasonal variations in the pigments. Chlorophyll was more abundant during summer than in the other two seasons. During this period, the intensity of light on the thalli during low tide may also influence the production of pigment during summer [64]. The presence of photosynthetic pigments may help in the absorption of light and energy in the reaction center and may also involve protection against light stress [8,65,66]. The increased level of carotenoid during monsoon could be a stress response to protect the photosynthetic apparatus against environmental stress [8,67]. The variation in the concentration of different pigments in response to environmental changes may help the seaweed to adapt to the seasonality in the rocky intertidal habitat.

Metabolic activities are altered by abiotic factors such as temperature, pH, and nutrient availability according to environmental conditions, causing changes in the biochemical compositions of green seaweed *C. antennina* [68]. Sugars and proteins are the most important components for various processes and for the endurance of seaweeds under changing environmental conditions in coastal systems. Photosynthetic pigments utilize sunlight for the production of carbohydrates through the process of photosynthesis. Therefore, we measured the concentration of carbohydrates to understand the biochemical response to various seasons. Carbohydrates are among the vital sources responsible for providing energy for respiration and other metabolic processes in seaweeds [69]. Significant variations in various monosaccharides were observed in different seasons. The highest concentrations of individual sugars, including glucose, xylose, and total sugars, were observed during the post-monsoon season. Galactose, rhamnose, and arabinose levels were higher during the monsoon period. Thus, from this result, we could suggest based on seasonality that green alga *C. antennina* can be seen as a source of dietary fibers, safeguarding the potentially reduced digestibility that might compromise these potential benefits [70].

### 4.2. Seasonal Changes Altered the Primary Metabolism of C. antennina

Generally, fatty acids are susceptible to changes in the environmental conditions and also play an important role in algal physiology [71]. Seaweeds contain limited concentrations of fatty acids in their tissue compared with all other metabolites, but their needs are significant in terms of nutritional properties [12]. Monsoon and post-monsoon have been shown to cause higher concentrations of fatty acids, indicating nutritionally rich contents during these seasons, and could be the apt periods for harvesting seaweeds for fatty acids. Our result is in line with previous findings on another green alga, *U. lactuca* [69], and economically important brown algae [12]. Seasonal variations in the levels of the fatty acids may be due to higher temperatures during summer, as Sánchez-Machado et al. [72] also showed, indicating that at higher temperatures, lipid concentrations may decrease or stay stable until favorable conditions are reached. The increase in PUFAs during monsoon may happen because the cell membrane tends to increase in tightness due to lower temperatures [70]. Temperatures are also known to affect the fatty acid concentrations in the cell membrane. PUFAs also act as electron carriers in the photosynthetic mechanisms of seaweeds for the production of energy [73]. PUFAs are important components of many invertebrates’ dietary needs [74]. Increased concentrations of PUFAs during monsoon also increase the nutritional quality of *C. antennina*. In addition, the concentrations of SFAs were found to be increased in post-monsoon. SFAs can be best harvested during this period and are known to have important applications for human health because of the importance of C14:0 and C16:0 for the synthesis of cholesterol [70].

Amino acids serve as substitutes for carbohydrates for the energy requirement under abiotic stress conditions [73]. Most AAs in our study were at somewhat higher levels in post-monsoon than in summer and monsoon. AAs can also vary depending on seasonality [69]. A similar kind of observation was also found for Chlorophyta *Ulva lactuca* from Egypt, with this species having higher concentrations during the spring season [69]. Proline is one of the luxuriant AAs present in *C. antennina* throughout the year. Some of the amino acids, including proline, act as protective osmolytes under saline conditions [1,75]. Therefore, *C. antennina* possesses higher nutritional qualities in terms of FAs and AAs during monsoon and post-monsoon season, and we find this season to be an apt period for harvesting the alga in terms of economic aspects.

### 4.3. Seasonal Changes Altered the Antioxidant Properties of C. antennina

Intertidal rocky green seaweed is subjected to some harsh environmental conditions such as desiccation conditions, heat stress, high light exposure, and carbon and nutrient limitations. These extreme conditions can induce the formation of ROS and contribute to photoinhibition processes [76]. These ROS could disrupt or inactivate the enzymes involved in the metabolic process through oxidative damage to DNA, RNA, and proteins [76]. Significant variations in antioxidant activity may be due to the environmental parameters of different seasons. Farasat et al. [77] also showed that the time of collection also mattered in the antioxidant activity of *C*. *aerea*. The levels of antioxidant molecules and enzymes were higher during the post-monsoon period than in monsoon and summer. The increased antioxidant status of *C. antennina* could help the algae to resist the abiotic stress factors due to environmental changes [6]. An increase in phenolic and flavonoid contents was also observed in post-monsoon. This may have been due to the fact that polyphenols may contribute to the major portion of total antioxidant content. A similar kind of increase in the contents of polyphenols and flavonoids was observed in a previous study from Iran on *C. linum* and *C. aerea* [77]. Flavonoids are generally found in epidermal cells and have the ability to absorb UV light. In an aquatic environment, quercetin can be induced by UV-B and functions as a UV screen [78,79]. At low temperatures, antioxidant enzymes increase to cope with antioxidant stress [73]. According to Wu et al. [80], a higher antioxidant activity of seaweeds was due to the higher content of antioxidant molecules such as ascorbate, glutathione, phenols, and flavonoids. CAT and POX are antioxidant enzymes enacting a defense mechanism that prevents algal tissue from lipid peroxidation, protein denaturation, nucleic acid damage, and pigment loss [8]. The accumulated ROS may be removed by CAT and POX. These enzymes are present in higher concentrations during the post-monsoon season to cope with physiological stress in algae [8,81]. Antioxidant activity is an important factor that indicates that the physiological stress in seaweeds is influenced by seasonal variations. Thus, understanding the yearly pattern of the trend followed by the nutritional and antioxidant properties of seaweeds may help to understand the importance of seaweeds, and further research would be helpful to obtain a better understanding of how other associated communities benefit from algae and to observe the pattern in the food web.

### 4.4. Variations in Minerals and Trace Elements

An increase in the contents of various essential minerals during post-monsoon in *C. antennina*, including iron (Fe), calcium (Ca), sodium (Na), magnesium (mg), manganese (Mn), selenium (Se), etc., could be a possible source of dietary need. The concentrations of minerals also determine the quality of seaweed [82,83]. Fe is involved in many metabolic activities, such as the transportation of electrons and oxygen; Mn is involved in amino acid, fatty acid, and carbohydrate metabolism and also acts as a cofactor of many enzymes, including SOD, arginase, and pyruvate carboxylase [84]. The richness of these minerals also makes seaweed commercially important due to its enhanced nutritional properties.

## 5. Conclusions

The present study revealed prominent seasonal variations in the physiological and biochemical composition of *C. antennina*. Seaweeds are becoming more important as resources due to their ecological and commercial importance. Productivity and the levels of photosynthetic pigments were the highest during summer, while antioxidants, biochemical components (including amino acids), and the contents of minerals in *C. antennina* were mostly enhanced during post-monsoon. However, sugars and fatty acids were at their highest levels during the monsoon period. These variations may have been due to physiological adaptation to environmental conditions. To conclude, based on seasonality, post-monsoon may be the right choice for harvesting nutritionally rich seaweeds. However, in the future, further studies would also need to be conducted to gain more insights into the impact of various seasons on the ecological pattern associated with green algae.

## Figures and Tables

**Table 1:** Seasonal variations in the concentrations (mg/g) of various fatty acids in *C. antennina*. Saturated fatty acids, SFAs (A); mono unsaturated fatty acids, MUFAs (B); poly unsaturated fatty acids, PUFAs (C). Values are shown as means ± S.E. (*n* = 3). Different letters show significance, *p* < 0.05 (a = summer to monsoon; b = summer to post-monsoon; c = monsoon to Post monsoon).

	Summer	Monsoon	Post-Monsoon
**C12:0**	**0.289 ± 0.023 ^ab^**	**0.573 ± 0.036 ^a^**	**0.562 ± 0.084 ^b^**
**C14:0**	**0.011 ± 0.001 ^a^**	**0.022 ± 0.002 ^ac^**	**0.014 ± 0.002 ^c^**
**C15:0**	0.017 ± 0.002	0.031 ± 0.002	1.565 ± 1.543
**C16:0**	8.481 ± 0.623	10.806 ± 0.536	11.742 ± 2.291
**C16:1**	**0.079 ± 0.005 ^ab^**	**0.128 ± 0.008 ^a^**	**0.121 ± 0.017 ^b^**
**C16:2**	**0.007 ± 0.001 ^a^**	**0.012 ± 0.001 ^ac^**	**0.008 ± 0.001 ^c^**
**C16:3**	0.007 ± 0.001	0.013 ± 0.001	0.049 ± 0.04
**C17:0**	**0.225 ± 0.02 ^a^**	**0.32 ± 0.021 ^a^**	0.253 ± 0.04
**C17:1**	0.048 ± 0.005	0.087 ± 0.004	0.187 ± 0.128
**C18:0**	**0.751 ± 0.062 ^ab^**	**1.306 ± 0.084 ^a^**	**1.191 ± 0.061 ^b^**
**C18:1**	**3.244 ± 0.251 ^a^**	**6.378 ± 0.649 ^ac^**	**4.337 ± 0.713 ^c^**
**C18:2**	**0.185 ± 0.022 ^a^**	**0.328 ± 0.019 ^ac^**	**0.198 ± 0.032 ^c^**
**C18:3**	**0.033 ± 0.002 ^a^**	**0.059 ± 0.005 ^ac^**	**0.039 ± 0.006 ^c^**
**C20:0**	0.018 ± 0.004	0.03 ± 0.004	0.017 ± 0.003
**C20:2**	**0.02 ± 0.002 ^a^**	**0.037 ± 0.004 ^ac^**	**0.024 ± 0.004 ^c^**
**C22:0**	**0.011 ± 0.001 ^a^**	**0.02 ± 0.001 ^a^**	0.016 ± 0.002
**C22:2**	**0.016 ± 0.002 ^ab^**	**0.029 ± 0.002 ^a^**	**0.024 ± 0.002 ^b^**
**C23:0**	**0.007 ± 0.001 ^ab^**	**0.016 ± 0.004 ^a^**	**0.024 ± 0.002 ^b^**
**C24:0**	**0.011 ± 0.001 ^b^**	0.02 ± 0.002	**0.034 ± 0.01 ^b^**
**C24:1**	**0.039 ± 0.002 ^ab^**	**0.07 ± 0.007 ^a^**	**0.08 ± 0.006 ^b^**
**C25:0**	**0.03 ± 0.002 ^ab^**	**0.061 ± 0.007 ^a^**	**0.055 ± 0.007 ^b^**
**C26:0**	**0.0008 ± 0.0001 ^b^**	0.0014 ± 0.0001	**0.0018 ± 0.0001 ^b^**

**Table 2:** Seasonal variations in the concentration (µg/g) of various mineral of *C. antennina*. Values are shown as means *±* S.E. (*n* = 3). Different letters show significance, *p* < 0.05 (a = summer–monsoon; b = summer–post-monsoon; c = monsoon–post-monsoon).

	Summer	Monsoon	Post-Monsoon
**Na**	18453.2 ± 509.5	**17262.2 ± 1170.6 ^c^**	**20336.1 ± 567.3 ^c^**
**Rb**	4.7 ± 0.2	**4.5 ± 0.4 ^c^**	**5.4 ± 0.2 ^c^**
**Sr**	**897.2 ± 24.8 ^ab^**	780.7 ± 21.2 ^ac^	**1091.3 ± 30 ^bc^**
**Cd**	0.283 ± 0.009	**0.268 ± 0.021 ^c^**	**0.319 ± 0.01 ^c^**
**In**	0.37 ± 0.02	0.36 ± 0.04	0.45 ± 0.02
**Cs**	0.53 ± 0.01	**0.5 ± 0.04 ^c^**	**0.59 ± 0.02 ^c^**
**Ba**	79.4 ± 3.6	77.4 ± 8.5	96.5 ± 3
**Pb**	5.2 ± 0.3	5.1 ± 0.6	6.4 ± 0.3
**Bi**	0.14 ± 0.01	0.13 ± 0.01	0.16 ± 0.01
**Mg**	**2917.6 ± 88.7 ^b^**	**2973.9 ± 45.6 ^c^**	**3280.9 ± 92.7 ^bc^**
**Al**	**97.7 ± 5.1 ^ab^**	**136.5 ± 10.3 ^a^**	**144.1 ± 4.5 ^b^**
**Ca**	3600.9 ± 104.6	**3400.5 ± 262.4 ^c^**	**4058.5 ± 110.5 ^c^**
**V**	1.2 ± 0.05	1.15 ± 0.11	1.41 ± 0.05
**Cr**	9.9 ± 0.3	8.9 ± 0.3	9.9 ± 0.3
**Mn**	62.1 ± 1.7	**57.8 ± 3.7 ^c^**	**67.7 ± 1.9 ^c^**
**Fe**	182.5 ± 5.8	**173.1 ± 14.2 ^c^**	**207.7 ± 6 ^c^**
**Co**	0.29 ± 0.01	0.28 ± 0.03	0.36 ± 0.01
**Ni**	2.6 ± 0.1	2.4 ± 0.2	2.8 ± 0.1
**Cu**	7.3 ± 0.2	6.6 ± 0.3	7.5 ± 0.2
**Zn**	72.4 ± 3	70.2 ± 7.4	87 ± 2.7
**Ga**	0.063 ± 0.002	**0.059 ± 0.005 ^c^**	**0.071 ± 0.002 ^c^**
**K**	288 ± 13.6	**268.8 ± 23.3 ^c^**	**330.2 ± 8.3 ^c^**
**As**	25.5 ± 0.7	**24 ± 1.8 ^c^**	**28.5 ± 0.8 ^c^**
**Se**	4.9 ± 0.2	**4.7 ± 0.4 ^c^**	**5.7 ± 0.2 ^c^**

## Data Availability

Not applicable.

## Supplementary Materials

The following supporting information can be downloaded at: https://www.mdpi.com/article/10.3390/biom12101475/s1, Supplementary Table S1: Water quality parameters at the time of sample collection. Averages ± SE values are given in the table, Supplementary Table S2: Monthly variations in the carbohydrate contents in *C. antennina*. Averages ± SE values are given in the table, Supplementary Table S3: Monthly variations in the amino acid contents in *C. antennina*. Averages ± SE values are given in the table, Table S4: Monthly variations in the fatty acid contents in *C. antennina*. Averages ± SE values are given in the table, Supplementary Table S5: Monthly variations in mineral contents in *C. antennina*. Averages ± SE values are given in the table, Supplementary Table S6: Monthly variations in the level of the antioxidant contents in *C. antennina*. Averages ± SE values are given in the table.
