# Supplementary material for: Seasonal Changes in the Biochemical Constituents of Green Seaweed Chaetomorpha antennina from Covelong, India
Source: Biomolecules. 2022 Oct 13;12(10):1475. doi: 10.3390/biom12101475 (PMC9599584; doi:10.3390/biom12101475)
Supplement: Supplementary file 1 [file biomolecules-12-01475-s001.zip › biomolecules-1896861-supplementary.pdf]

Supplementary files

Table S1. Water quality parameters at the time of sample collection in the study site. Values are average ±SE values are given in table, n= 3.

	Light (LUX)	Temperature (°C)	DO (mL/L)	pH	Salinity (PPT)	Ammonia (µM)	Nitrite (µM)	Nitrate (µM)
19/6/2019	530100±115000	29.4±0.2	7.7±0.1	8.13±0.03	33.1±0.1	0.06±0.01	0.02±0	0.06±0.03
04/07/2019	476100±12000	28.8±0.3	5.8±0.1	8.15±0.02	32.6±0.1	0.04±0.01	0.02±0	0.92±0.04
02/08/2019	365100±1000	28.9±0.2	7.7±0.1	8.14±0.05	33.45±0.05	0.2±0.01	0.01±0	1.38±0.08
27/09/2019	638600±198500	29.1±0.1	7±0.3	8.12±0.02	32.8±0.2	0.23±0.03	0.05±0.01	0.78±0.07
31/10/2019	372600±102500	29.1±0.3	6.9±0	7.98±0.02	30.75±0.25	0.015±0.009	0.008±0.003	0.42±0.142
12/11/2019	341600±270500	28.4±0.2	6.6±0.3	8.07±0.01	30.2±0.2	0.02±0.004	0±0.002	0.83±0.083
12/12/2019	619600±198500	27.1±0.2	6.9±0	7.98±0.03	29.35±0.15	0.02±0.002	0.01±0.002	0.45±0.07
29/01/2020	886100±289000	26.9±0.2	7.2±0.2	8.1±0.04	29.9±0.1	0.42±0.01	0.04±0.01	1.31±0.07
22/02/2020	926600±29500	27.4±0.2	4.7±0.1	8.14±0.05	30.15±0.15	0.52±0.07	0.02±0.01	1.97±0.15
01/03/2020	806600±170500	28.3±0.2	7.4±0	8.12±0.08	31.9±0.1	0.68±0.01	0.07±0.01	2.76±0.04

Measurement of Physico-chemical parameters: At the time of sample collection, the light was measured using a Lux meter (Lutron LX 101A) and, temperature, pH, salinity, dissolved oxygen were measured using a combo water quality meter (AZ 86031, M&A instruments, USA). Nitrate concentration in the water samples was measured through a spectrophotometer using standard procedures.

Table S2. Month wise variations in the carbohydrate content of *C. antennina*. Values are average ±SE values are given in table, n= 3.

	June'19	July'19	Aug'19	Sep'19	Oct'19	Nov'19	Dec'19	Jan'20	Feb'20	Mar'20
**Rhamnose**	27.29±0.24	33.1±1.05	35.4±3.05	46.09±3.15	39.78±1.47	50.93±1.05	57.53±0.93	52.34±0.51	42.54±1.33	49.83±1.26
**Arabinose**	0.04±0	0.05±0	0.05±0	0.07±0.01	0.08±0.01	0.1±0	0.12±0.01	0.08±0	0.09±0.01	0.03±0.01
**Galactose**	3.73±0.04	3.98±0.17	4.95±0.15	7.12±1.1	7±0.2	7.92±1	9.77±0.32	7.07±0.08	7.4±0.26	7.12±1.1
**Glucose**	49.57±2.09	45.42±2.84	54.47±3.12	70.05±12.7	66.73±4.57	74.35±4.72	62.02±6.87	77.29±7.81	96.04±8.89	80.1±5.06
**Xylose**	16.38±1.56	18.26±0.4	23.67±1.81	25.86±0.36	30.98±3.22	32.71±3.68	35.2±0.89	38.18±3.75	34.84±1.04	43.86±2.77
**Glucuronic acid**	13.87±0.76	12±0.73	18.35±2.09	28.3±3.03	16.36±0.19	26.79±1.74	22±0.63	24.4±2.01	28.35±3.45	21.31±0.26
**Total**	106.17±4.46	97.46±6.01	116.96±6.54	150.18±27	157.72±11.35	159.84±10.26	134.22±14.51	151.68±21.9	172.53±15.9	170.82±10.77

Table S3. Month wise variations in the amino acid content of *C. antennina*. Values are average ±SE values are given in table, n= 3.

	**June'19**	**July'19**	**Aug'19**	**Sep'19**	**Oct'19**	**Nov'19**	**Dec'19**	**Jan'20**	**Feb'20**	**Mar'20**
**Proline**	114±15.23	179.17±7.45	220.17±5.42	156.99±7.31	122.54±27.79	112.95±22.1	154.28±22.32	184.21±10.29	195.08±5.48	187.68±12.76
**Glycine**	7.61±0.16	8.64±0.7	8.94±0.24	8.34±0.24	8±1.2	7.25±1.5	7.87±1.84	7.74±1.66	10.17±1.79	13.93±0.96
**Serine**	6.49±0.17	6.12±0.57	6.59±0.18	6.95±0.18	6.11±1.1	5.99±1.42	5.9±1.54	6.17±1.17	8.07±1.68	7.93±1.01
**Argnine**	29.76±1.64	45.55±1.77	30.54±2.71	21.14±0.59	16.84±5.28	27.41±5.66	36.66±4.65	27.75±4.88	29.5±7.29	42.71±4.32
**Ornithine**	173.38±13.93	248.86±12.37	152.35±8.5	145.14±7.65	100.42±29.59	158.62±30.27	205.16±28.14	153.32±26.84	172.7±39.57	242.52±24.96
**Glutamine**	282.09±11.3	399.88±13.29	262.79±22.3	229.82±5.19	244.59±1.66	435.67±31.16	395.22±48.5	397.33±28.1	453.51±21.17	553.93±19.48
**Glutamate**	117.14±0.21	106.42±9.2	95.72±4.74	105.02±2.14	121.59±9.85	128.83±4.72	158.56±8.45	161.33±17.07	166.95±6.87	207.1±8.7
**Aspartate**	6.28±0.35	5.17±1.01	22.34±16.13	26.55±17.67	7.69±1.34	7.72±0.62	6.54±1.87	7.72±0.93	10.27±1.22	9.51±1.01
**Cystine**	0.22±0.11	0.47±0.07	0.06±0.01	0.07±0.01	0.05±0.01	0.15±0.06	0.29±0.04	0.24±0.02	0.14±0.05	0.3±0.05
**Asparagine**	0.1±0.001	0.11±0.01	0.03±0.003	0.1±0.01	0.09±0.02	0.09±0.01	0.09±0.01	0.11±0.01	0.12±0.02	0.12±0.01
**Leucine**	0.06±0.02	0.07±0.003	0.01±0.002	0.12±0.01	0.08±0.02	0.07±0.01	0.29±0.21	0.29±0.22	0.1±0.01	0.24±0.14
**Lysine**	0.26±0.02	0.2±0.01	0.05±0.001	0.31±0.01	0.25±0.05	0.25±0.06	0.22±0.06	0.3±0.02	0.34±0.07	0.32±0.04
**Histidine**	0.22±0.01	0.15±0.01	0.05±0.0003	0.27±0.004	0.22±0.04	0.21±0.04	0.17±0.04	0.2±0.04	0.29±0.05	0.26±0.03
**Alanine**	1.15±0.4	2.11±0.24	0.3±0.05	0.59±0.05	0.47±0.11	0.86±0.28	1.41±0.21	1.23±0.1	0.88±0.26	1.51±0.21
**Isoleucine**	0.63±0.05	0.68±0.03	0.16±0.01	0.88±0.09	0.68±0.13	0.63±0.08	0.64±0.11	0.63±0.16	0.88±0.14	0.85±0.06
**Methionine**	2.04±0.39	1.52±0.2	0.44±0.02	2.66±0.08	2.15±0.42	1.95±0.32	3.39±1.75	1.87±0.37	2.73±0.49	3.56±0.98
**Threonine**	4.04±0.14	2.8±0.17	0.69±0.01	4.32±0.15	3.51±0.67	5.44±0.85	3.18±0.88	3.45±0.61	5.97±0.13	5.75±0.96
**Valine**	4.69±1.41	15.3±2.08	2.54±0.36	6.07±0.74	4.96±0.95	4.49±0.78	10.69±2.14	9.96±0.43	6.3±0.75	10.12±0.91
**Phenylalanine**	7.37±0.26	15.82±1.62	2.48±0.26	7.82±0.41	6.16±1.33	6.84±1.18	11.63±1.99	8.78±2.29	8.67±1.67	12.31±0.89
**Tyrosine**	11.32±1.97	9.86±0.85	1.11±0.11	15.74±0.53	12.36±2.3	27.43±14.68	12.09±1.08	12.83±1.25	26.52±8.34	25.26±10.18

Table S4. Month wise variations in the fatty acids content of *C. antennina*. Values are average ±SE values are given in table, n= 3.

	June'19	July'19	Aug'19	Sep'19	Oct'19	Nov'19	Dec'19	Jan'20	Feb'20	Mar'20
**Dodecanoic (C12:0)**	0.21±0.002	0.25±0.008	0.3±0.009	0.4±0.043	0.43±0.016	0.66±0.006	0.63±0.01	0.78±0.02	0.46±0.014	0.44±0.221
**Tetradecanoic (C14:0)**	0.01±0.0003	0.01±0.001	0.01±0.001	0.02±0.001	0.02±0.002	0.02±0.001	0.03±0.003	0.02±0.001	0.02±0.002	0.01±0.001
**Pentadecanoic (C15:0)**	0.01±0.0002	0.01±0.001	0.02±0.001	0.03±0.004	0.02±0.001	0.03±0	0.03±0.001	0.02±0.0003	0.03±0.001	4.64±4.633
**Hexadecanoic (C16:0)**	7.66±0.32	7.02±0.44	8.42±0.48	10.83±1.96	11.34±0.82	11.49±0.73	9.59±1.06	8.58±3.6	14.84±1.37	11.8±6.19
**Hexadecanoic (C16:1)**	0.06±0.006	0.07±0.002	0.09±0.007	0.1±0.001	0.13±0.01	0.13±0.023	0.12±0.002	0.12±0.012	0.13±0.004	0.12±0.058
**Hexadecadienoic (C16:2)**	0.01±0.0003	0±0.0003	0.01±0.001	0.01±0.002	0.01±0.0001	0.01±0.002	0.01±0.0004	0.01±0.001	0.01±0.001	0.004±0.001
**Hexadecatrienoic (C16:3)**	0.01±0.0002	0.01±0.0001	0.01±0.0002	0.01±0.002	0.01±0.0001	0.01±0.001	0.01±0.0002	0.01±0.0003	0.01±0.0002	0.13±0.122
**Heptadecanoic (C17:0)**	0.18±0.01	0.16±0.003	0.24±0.016	0.31±0.05	0.33±0.02	0.36±0.036	0.27±0.036	0.34±0.019	0.21±0.055	0.21±0.099
**heptadecenoic (C17:1)**	0.04±0.001	0.04±0.001	0.05±0.002	0.07±0.012	0.07±0.002	0.1±0.001	0.1±0.002	0.07±0.001	0.07±0.001	0.42±0.39
**Octadecanoic (C18:0)**	0.61±0.02	0.59±0.001	0.78±0.08	1.03±0.13	1.05±0.08	1.42±0.13	1.45±0.11	1.16±0.04	1.1±0.01	1.31±0.18
**Octadecenoic (18:1)**	3.12±0.14	2.64±0.28	3.11±0.46	4.11±0.71	4.32±0.81	7.55±0.7	7.26±0.84	5.81±0.25	5.52±0.25	1.68±0.81
**Octadecadienoic (C18:2)**	0.15±0.002	0.13±0.00004	0.19±0.004	0.28±0.061	0.26±0.007	0.38±0.0004	0.34±0.007	0.27±0.003	0.24±0.002	0.08±0.034
**Octadecatrienoic (C18:3)**	0.03±0.003	0.03±0.001	0.04±0.002	0.04±0.002	0.05±0.002	0.05±0.014	0.07±0.003	0.05±0.005	0.05±0.001	0.02±0.006
**Eicosanoic (C20:0)**	0.01±0.002	0.01±0.001	0.01±0.001	0.03±0.011	0.02±0.001	0.05±0.006	0.03±0.001	0.03±0.003	0.02±0.001	0.01±0.002
**Eicosadienoic (C20:2)**	0.01±0.0004	0.02±0.0002	0.02±0.002	0.03±0.003	0.03±0.005	0.04±0.002	0.04±0.008	0.03±0.001	0.03±0.001	0.01±0.003
**Docosanoic (C22:0)**	0.01±0.0004	0.01±0.0002	0.01±0.001	0.01±0.001	0.02±0.002	0.02±0.002	0.02±0.003	0.02±0.001	0.02±0.001	0.02±0.008
**Docosenoic (22:2)**	0.01±0.0001	0.01±0.0002	0.01±0.001	0.03±0.002	0.02±0.004	0.03±0.0003	0.03±0.005	0.02±0.002	0.02±0.003	0.03±0.003
**Tricosanoic (C23:0)**	0.004±0.0001	0.01±0.002	0.01±0.0002	0.01±0.002	0.02±0.008	0.01±0.0002	0.02±0.01	0.02±0.002	0.02±0.003	0.03±0.003
**Tetracosanoic (C24:0)**	0.01±0.001	0.01±0.001	0.01±0.001	0.02±0.002	0.02±0.002	0.02±0.003	0.02±0.003	0.02±0.002	0.02±0.003	0.06±0.03
**Tetracosenoic (C24:1)**	0.03±0.003	0.04±0.001	0.04±0.005	0.05±0.0001	0.06±0.009	0.07±0.011	0.08±0.013	0.07±0.006	0.08±0.01	0.09±0.012
**Pentacosanoic (C25:0)**	0.02±0.002	0.04±0.0001	0.03±0.003	0.03±0.007	0.05±0.008	0.05±0.004	0.08±0.012	0.06±0.004	0.06±0.007	0.04±0.02
**Hexacosanoic (26:0)**	0.001±0.00003	0.001±0.000001	0.001±0.0001	0.001±0.0004	0.001±0.0001	0.002±0.0001	0.001±0.00002	0.002±0.0002	0.002±0.00003	0.002±0.00

Table S5. Month wise variations in the mineral content of *C. antennina*. Values are average ±SE values are given in table, n= 3.

	June'19	July'19	Aug'19	Sep'19	Oct'19	Nov'19	Dec'19	Jan'20	Feb'20	Mar'20
**Na**	16644.69±119.29	17270.55±93.62	20708.71±472.87	19189.03±560.61	21830.68±197.94	14131.35±85.83	15824.69±164.75	18998.92±489.94	19533.2±294.64	22476.27±168.66
**Rb**	4.19±0.07	4.32±0.08	5.2±0.26	5.15±0.24	5.89±0.09	3.54±0.06	3.96±0.14	4.93±0.24	5.25±0.16	5.89±0.09
**Sr**	837.14±6.92	860.49±7.74	1036.15±14.82	855.12±13.05	844.34±24	707.35±6.09	790.27±2.96	995.84±13.01	1079.11±5.33	1198.97±13.35
**Cd**	0.25±0.01	0.26±0.01	0.31±0.01	0.31±0.01	0.35±0.01	0.21±0	0.24±0	0.29±0.01	0.31±0.01	0.35±0.01
**In**	0.32±0.01	0.32±0.01	0.39±0.01	0.44±0.02	0.51±0.02	0.27±0.01	0.3±0.01	0.4±0.02	0.46±0.02	0.49±0.02
**Cs**	0.47±0	0.49±0	0.59±0.01	0.56±0.01	0.64±0.01	0.4±0	0.45±0.01	0.55±0.01	0.57±0	0.65±0.01
**Ba**	68.23±0.5	69.26±0.68	84.05±2.76	95.94±3.28	110.96±1.02	57.29±0.49	63.88±1.3	85.71±2.84	98.52±2.03	105.15±0.66
**Pb**	4.39±0.16	4.44±0.17	5.39±0.36	6.4±0.46	7.44±0.36	3.68±0.14	4.1±0.22	5.62±0.39	6.59±0.39	6.95±0.3
**Bi**	0.12±0.01	0.13±0.01	0.15±0.01	0.15±0.01	0.17±0.01	0.1±0.01	0.12±0.01	0.14±0.01	0.15±0.01	0.17±0.01
**Mg**	2606.6±27.04	2693.23±26.31	3238.13±122.48	3132.49±93.77	2908.71±21.45	2874.93±20.19	3138.07±59.91	3033.63±102.97	3194.22±42.17	3614.77±24.01
**Al**	84.98±1.12	94.64±0.07	124.47±1.29	86.88±7.63	116.3±25.38	143.49±1.63	149.64±17.83	127.6±1.13	147.52±2.11	157.05±2.89
**Ca**	3215.31±26.67	3320.71±33.85	3989.7±71.2	3877.69±79.34	4432.13±29.13	2723.34±25.16	3046±23.56	3746.38±71.47	3957.06±28.15	4472.17±33.93
**V**	1.05±0.02	1.07±0.02	1.29±0.06	1.38±0.08	1.59±0.04	0.88±0.02	0.98±0.03	1.27±0.07	1.42±0.06	1.54±0.03
**Cr**	9.32±0.08	9.84±0.14	11.68±0.07	8.78±0.17	9.79±0.09	7.98±0.09	8.97±0.03	9.74±0.11	8.84±0.07	11.11±0.11
**Mn**	56.24±0.43	58.47±0.66	70.02±2.4	63.5±0.99	72.11±1.31	47.8±0.35	53.54±1.23	63.58±1.55	64.57±0.45	74.94±0.73
**Fe**	162.26±2.37	167.26±4.14	201.08±7.72	199.47±5.6	228.49±2.55	137.3±2.56	153.47±4.86	190.74±6.3	203.79±3.79	228.62±3.19
**Co**	0.25±0.01	0.25±0.01	0.31±0.02	0.36±0.02	0.41±0.01	0.21±0	0.23±0.01	0.32±0.02	0.36±0.02	0.39±0.01
**Ni**	2.33±0.09	2.43±0.13	2.9±0.14	2.57±0.13	2.92±0.12	1.98±0.09	2.22±0.11	2.6±0.12	2.62±0.11	3.07±0.13
**Cu**	6.76±0.08	7.11±0.18	8.46±0.38	6.73±0.22	7.55±0.02	5.78±0.1	6.49±0.23	7.23±0.28	6.8±0.11	8.33±0.08
**Zn**	62.59±0.51	63.7±0.45	77.13±2.18	86.04±2.74	99.43±1.76	52.62±0.4	58.68±1.09	77.7±2.31	88.32±2.06	95.03±1.3
**Ga**	0.06±0	0.06±0	0.07±0	0.07±0	0.08±0	0.05±0	0.05±0	0.07±0	0.07±0	0.08±0
**K**	234.41±15.76	258.22±8.63	330.6±6.62	328.8±10.22	355.67±17.79	205.26±10.16	245.34±2.74	314±7.56	325.94±11.2	350.58±17.17
**As**	22.81±0.13	23.59±0.26	28.31±0.46	27.14±0.25	31±0.38	19.34±0.16	21.63±0.22	26.41±0.34	27.69±0.09	31.46±0.32
**Se**	4.24±0.05	4.34±0.06	5.24±0.22	5.59±0.23	6.43±0.1	3.58±0.05	3.99±0.12	5.16±0.22	5.72±0.16	6.25±0.08

Table S6. Month wise variations in the level of antioxidant content of *C. antennina*. Values are average ±SE values are given in table, n= 3.

	June'19	July'19	Aug'19	Sep'19	Oct'19	Nov'19	Dec'19	Jan'20	Feb'20	Mar'20
**GPX (µmol NADPH/mg protein/min)**	0.31±0.04	0.09±0	0.16±0.03	0.25±0.05	0.37±0.02	0.25±0.02	1.61±1.2	0.81±0.19	0.19±0.01	0.22±0.02
**GST (µmol/min/mg protein)**	0.28±0	0.24±0.04	0.27±0.02	0.28±0.01	0.2±0.01	0.21±0	0.23±0.03	0.21±0.03	0.17±0.06	0.34±0.06
**GR (µmol/min/mg protein)**	0.31±0.07	0.35±0.04	0.58±0.03	0.44±0.02	0.55±0.01	0.36±0.02	0.47±0.03	1.21±0.14	1.89±0.26	1.41±0.06
**DHAR (µmol/min/mg protein)**	0.46±0.09	0.39±0.12	0.49±0.08	0.43±0.03	0.5±0.08	0.37±0.1	0.48±0.03	0.51±0.1	0.77±0.1	0.81±0.19
**MDHAR (µmol/min/mg protein)**	0.17±0.03	0.22±0.01	0.19±0.04	0.13±0.02	0.17±0.02	0.19±0.03	0.13±0.03	0.21±0.06	0.13±0.04	0.17±0.04
**APX (µmol/min/mg protein)**	1.09±0.17	2.3±0.16	1.01±0.12	1±0.15	1.2±0.15	1.7±0.01	1.66±0.13	1.84±0.05	2.45±0.22	1.94±0.19
**CAT (µmol/min/mg protein)**	31.53±1.87	24.83±3.62	24±9.17	25.08±2.98	22.37±2.66	23.33±1.46	51.9±4.27	36.42±2.66	92.13±10.6	69±8.22
**POX (µmol/min/mg protein)**	1.73±0.18	1.52±0.32	1.55±0.31	1.4±0.28	1.64±0.12	1.75±0.29	2.73±0.28	3.17±0.45	3.61±0.41	4.13±0.44
**ASC (µmol/gFW)**	1.81±0.05	2.13±0.62	1.32±0.04	1.23±0.31	2.44±0.21	2.88±0.13	2.9±0.39	2.69±0.41	3.08±0.34	3.13±0.37
**TASC (µmol/gFW)**	3.34±0.51	2.89±0.82	2.14±0.22	2.24±0.46	3.77±0.5	4.79±0.56	4.05±0.11	6.38±1.07	5.94±0.32	5.93±0.96
**DHA (µmol/gFW)**	1.54±0.47	0.76±0.28	0.82±0.23	1.01±0.2	1.33±0.31	1.91±0.68	1.15±0.28	3.69±0.72	2.85±0.66	2.8±0.62
**GSH (µmol/gFW)**	0.19±0.05	0.28±0.07	0.11±0.02	0.17±0.06	0.26±0.07	0.29±0.05	0.24±0.04	0.4±0.06	0.5±0.07	0.37±0.05
**TGSH (µmol/gFW)**	0.33±0.08	0.34±0.08	0.15±0.02	0.24±0.06	0.38±0.02	0.37±0.1	0.28±0.04	0.55±0.08	0.65±0.13	0.57±0.16
**GSSG (µmol/gFW)**	0.14±0.03	0.06±0.03	0.04±0	0.07±0.01	0.13±0.07	0.08±0.06	0.05±0.01	0.15±0.03	0.15±0.07	0.2±0.12
**Polyphenol mg gallic acid/g FW**	11.61±4.4	12.63±3.4	12.65±4.07	8.39±2.71	11.18±3.24	26.62±3.33	13.13±0.38	23.21±0.35	20.36±6.54	22.23±6.35
**FRAP (µmol trolox/g FW)**	14.65±1.01	26.24±8.06	18.98±5.78	16.94±9.2	29.09±10.88	34.12±3.12	34.02±6.55	31.62±5.43	37.02±4.47	38.47±5.38
**MDA (nmol MDA/g FW)**	1.93±0.17	3.23±1.05	2.32±0.6	1.69±0.76	2.92±1.08	6.2±0.64	8.91±1.01	4.52±0.75	8.42±0.61	6.1±1.14

**Flavonoids (mg quercetin/g FW)**	0.21±0.01	0.26±0.08	0.19±0.05	0.18±0.05	0.23±0.06	0.4±0.08	0.38±0.03	0.34±0.04	0.47±0.01	0.46±0.05
**Tocopherols (mg/g FW)**	2±0.29	3±0.54	2.55±0.02	2.08±0.6	2.85±0.53	5.76±0.44	7.84±0.71	8.71±2.18	8.54±0.35	8.18±0.6
**SOD (unit/min/mg protein)**	840.98±111.35	466.08±187.77	632.61±99.51	822.8±52.72	548.11±87.05	630.07±182.24	1165.3±52.38	1075.84±90.7	1148.14±108.02	1193.87±3.78
